# Does Tourniquet Use Affect the Length of the Hamstring Tendon in Anterior Cruciate Ligament Reconstruction?

**DOI:** 10.3390/jcm15083076

**Published:** 2026-04-17

**Authors:** Tarık Altunkiliç, Bünyamin Ari, İsmail Güzel, Mehmet Boz, Feyza İnceoglu

**Affiliations:** 1 Department of Orthopedics and Traumatology, Faculty of Medicine, Malatya Turgut Ozal University, Malatya 44280, Turkey; bunyamin.ari@ozal.edu.tr (B.A.); ismail.guzel@ozal.edu.tr (İ.G.); mehmet.boz@ozal.edu.tr (M.B.); 2 Department of Biostatistics, Faculty of Medicine, Malatya Turgut Ozal University, Malatya 44280, Turkey; feyza.inceoglu@ozal.edu.tr

**Keywords:** anterior cruciate ligament reconstruction, hamstring tendon length, tourniquet

## Abstract

**Background:** The aim of the present study was to investigate whether the use of a tourniquet during anterior cruciate ligament reconstruction affects the length of the hamstring tendon used for autograft. **Methods:** Adult patients who were referred to the Orthopedics and Traumatology Department of The Malatya Turgut Özal University hospital and were diagnosed with anterior cruciate ligament injury between 30 July 2025 and 30 December 2025 were included in the study. In total, 31 patients from whom the hamstring tendon was harvested without a tourniquet were placed in Group 1, and 36 patients from whom the hamstring tendon was harvested with a tourniquet were placed in Group 2. This study is a prospective, randomized, comparative study. These two groups were compared with respect to the lengths of the hamstring tendons used as autografts. **Results:** The semitendinosus and gracilis tendon lengths in Group 1 were 29.61 ± 1.76 cm and 27.68 ± 2.02 cm, respectively. In Group 2, the semitendinosus and gracilis tendon lengths were 25.67 ± 1.45 cm and 23.72 ± 1.06 cm, respectively. A statistically significant difference was observed between the semitendinosus and gracilis tendon lengths of the participants in Groups 1 and 2 (*p* < 0.05). **Conclusions:** These findings suggest that avoiding tourniquet use during hamstring tendon harvest may represent a simple technical modification that can influence harvested tendon length during anterior cruciate ligament reconstruction.

## 1. Introduction

Available epidemiological data indicate that anterior cruciate ligament (ACL) injuries are relatively frequent, with an estimated incidence of approximately 68.6 cases per 100,000 person-years in the United States [1,2]. Therefore, orthopedists frequently perform ACL reconstruction. One of the most important steps in this surgery is to prepare the autograft or allograft to replace the ruptured ACL. Hamstring, quadriceps, Achilles, or bone–patellar tendon–bone (BPTB) tendons may all be used as autografts. Hamstring tendons (M. semitendinosus, M. gracilis) are a frequent choice; however, for these tendons to be turned into a strong graft for ACL reconstruction, the tendons must have an appropriate length and thickness. While there are many folding techniques available to reach the desired thickness of the autograft obtained from the harvested tendons, there is no clear technique by which the length of the tendon can be increased.

There have been numerous studies on the effects of tourniquet use on ACL reconstruction, but none on the effects of tourniquet use on tendon length. Because the tourniquet stops the bleeding in the surgical area, it improves the arthroscopic image [3]. The use of a tourniquet during an ACL reconstruction depends on the surgeon’s experience. Most orthopedists use tourniquets during hamstring tendon harvest. Some orthopedists harvest hamstring tendons without inflating the tourniquet and use them as autografts. Despite these two approaches, there are insufficient studies on whether tourniquet use affects tendon length. We aimed to investigate the potential effect of tourniquet use on tendon length during graft preparation.

Is there a surgical step that negatively impacts the length of the tendon during the operation? Does the tendon length increase after this stage is eliminated? There may be different answers to these two questions. However, we attempted to determine whether the use of a tourniquet was the appropriate response to these two problems. The aim of this study was to determine whether the length of the hamstring tendon changed when the tourniquet cuff was inflated (with tourniquet) or deflated (without tourniquet). Therefore, the aim of this prospective study was to evaluate the association between tourniquet use during hamstring tendon harvest and the harvested tendon length in patients undergoing ACL reconstruction.

## 2. Materials and Methods

**Figure 1:**
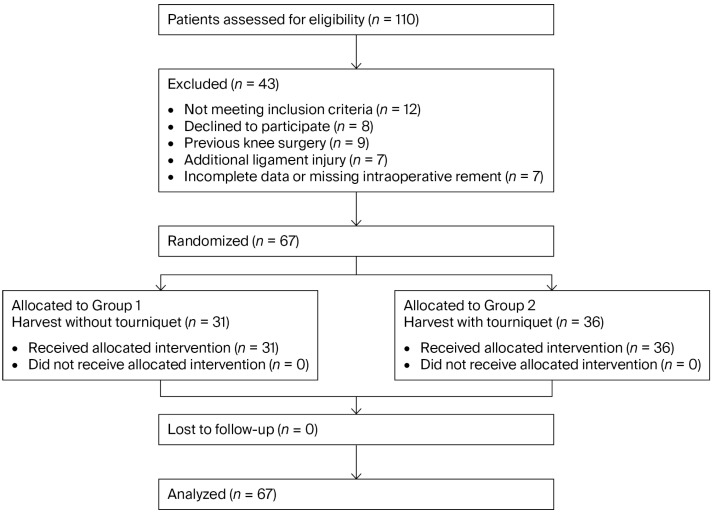
CONSORT flow diagram illustrating patient selection, randomization, and analysis of participants included in the study.

Adult patients who were referred to the Orthopedics and Traumatology Department of the Malatya Turgut Özal University hospital and were diagnosed with an ACL injury between 30 July 2025 and 30 December 2025 were included in the study. Patients who underwent tendon harvest with a tourniquet and those without were prospectively analyzed. All surgeries were performed by the same surgical team using a standardized anterior cruciate ligament reconstruction set. Written informed consent was obtained from each patient prior to group assignment. Allocation concealment was ensured using sequentially numbered, opaque, sealed randomization envelopes prepared by an independent investigator not involved in the patient recruitment or surgery. Randomization was performed using a computer-generated random number sequence prepared by an independent investigator who was not involved in the patient recruitment or surgery. Simple randomization was used with a 1:1 allocation ratio. The allocation sequence was concealed using sequentially numbered, opaque, sealed envelopes. After eligibility assessment and written informed consent, each patient was assigned to a study group by opening the next envelope in sequence. Randomization occurred prior to anesthesia induction and surgical skin preparation. No crossover between groups occurred after allocation. Of the 110 patients assessed for eligibility, 43 were excluded for the reasons detailed in the CONSORT flow diagram, and 67 patients were ultimately included and randomized (Figure 1). Data from all anterior cruciate ligament reconstruction cases were prospectively recorded by independent data collectors. Patients under 18 years of age and those with additional ligament injuries, interrupted postoperative follow-up, a history of previous surgery on the same knee, missing intraoperative tendon length measurements, and/or incomplete documentation were excluded from the study. Patients’ demographic and anthropometric characteristics, including age, sex, surgical side, body mass index (BMI), thigh circumference, and leg length, were recorded preoperatively. Hamstring tendon lengths were measured intraoperatively. Between 30 July 2025 and 30 December 2025, 110 patients were assessed for eligibility. To minimize anthropometric variability, patients with comparable BMI, thigh circumference, and leg length were considered eligible prior to group allocation. Following the eligibility assessment, 67 patients were prospectively included in the study. Among these patients, 31 underwent hamstring tendon harvest without tourniquet use (Group 1), and 36 underwent hamstring tendon harvest with tourniquet use (Group 2).

These two groups were compared with respect to the lengths of the hamstring tendons used as autografts. Leg length was measured as the distance between the spina iliaca anterior superior (SIAS) and the inferior border of the medial malleolus. The thigh circumference was measured 10 cm proximal to the superior pole of the patella. A wide, contoured pneumatic tourniquet (PT) was used for all patients, with the same tourniquet applied consistently across all procedures. The applied PT was a 15 cm wide contoured cuff. Before placing the tourniquet cuff, the most proximal thigh was wrapped in three layers of gypsum cotton. Thereafter, the tourniquet cuff was tightly wrapped by the surgeon and the assistant, extending it to the most proximal part of the thigh. The tourniquet pressure applied to the patients was twice the systolic pressure. Preoperative consent forms were completed by all patients, and the necessary information was provided. The patients were informed about the study, and their written consent was obtained. The Faculty of Medicine’s ethics committee granted the study permission on 26 June 2025 (Decision number 279722325). The Declaration of Helsinki’s ethical principles were upheld throughout the entire study’s processes.

Hamstring tendons consisting of semitendinosus and gracilis tendons were used as grafts in all 67 patients. For all of the patients, an EndoButton implant was used for femoral fixation of the graft. The tibia was fixed using a hybrid fixation that included a bioabsorbable screw and a washer screw or staple (U nail). The lengths of the tendons harvested from the patients during the procedure were measured and recorded.

**Figure 2:**
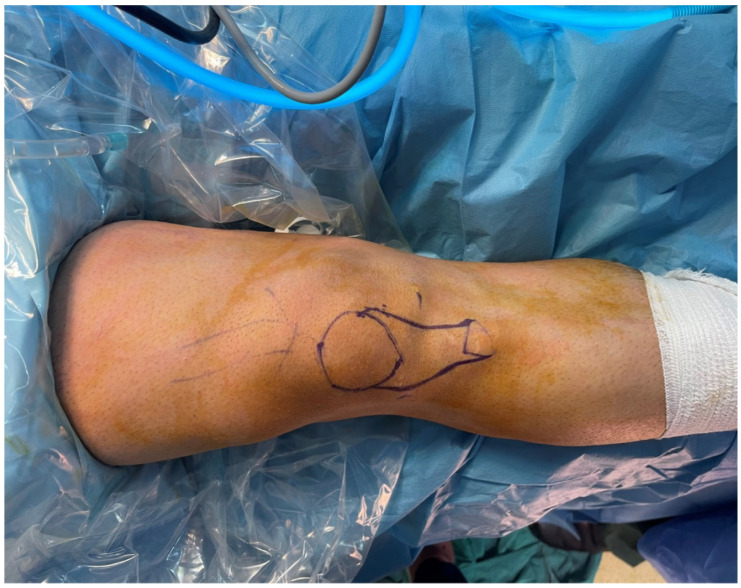
Preoperative knee preparation and marking of anatomical landmarks for hamstring tendon harvest.

**Figure 3:**
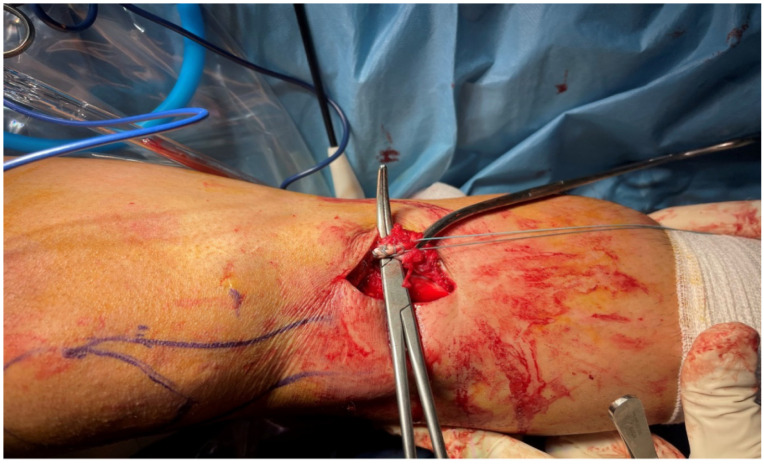
Preparation and Krackow suturing of the semitendinosus and gracilis tendons before harvesting.

A tourniquet cuff was applied to the lower limb in all patients (Figure 2). However, in Group 1, the cuff was not inflated during hamstring tendon harvesting, and the tendon harvest was performed without tourniquet inflation. In Group 2, the tourniquet was inflated before arthroscopy and remained inflated during the tendon-harvesting procedure. In Group 1, medial and lateral arthroscopic portals were opened prior to tourniquet inflation, and anterior cruciate ligament rupture was confirmed before the tourniquet cuff was inflated. In Group 2, the tourniquet cuff was inflated before arthroscopy, after which medial and lateral portals were opened and ACL rupture was confirmed. In both groups, hamstring tendons were identified through a 2 cm anteromedial oblique incision made 1.5 cm medial to the tibial tubercle. The ends of the semitendinosus and gracilis tendons were detached from the bone and sutured using the Krackow technique with No. 2 reinforced sutures (Figure 3). All lateral fascial bands and ligamentous structures restraining the tendons were carefully released until complete tendon liberation was achieved. The hamstring tendons were then harvested using a tendon stripper.

**Figure 4:**
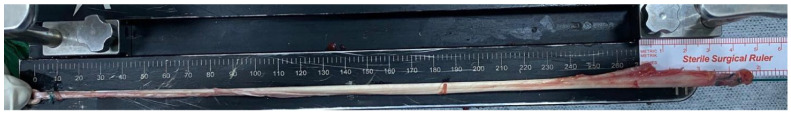
Intraoperative measurement of harvested hamstring tendon length.

**Figure 5:**
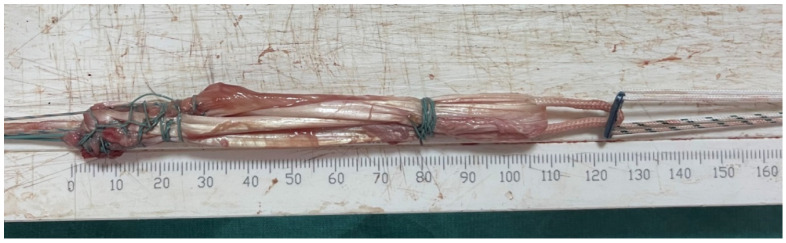
Preparation of the hamstring autograft after tendon harvest, demonstrating graft folding and length measurement.

**Figure 6:**
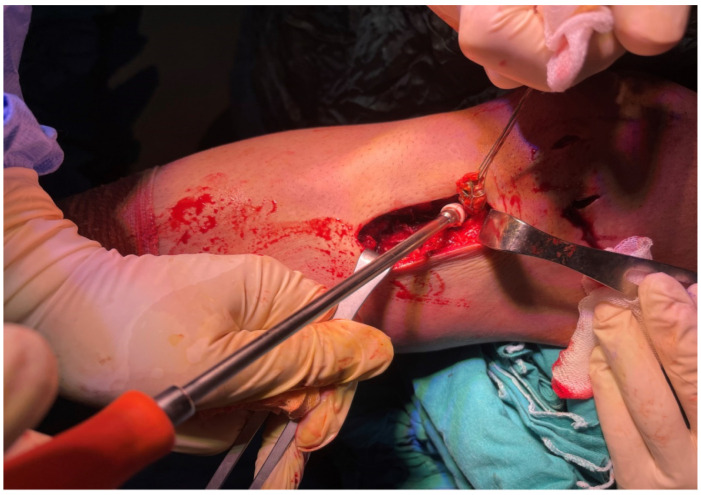
Tibial fixation of the hamstring autograft using a bioabsorbable interference screw during anterior cruciate ligament reconstruction.

**Figure 7:**
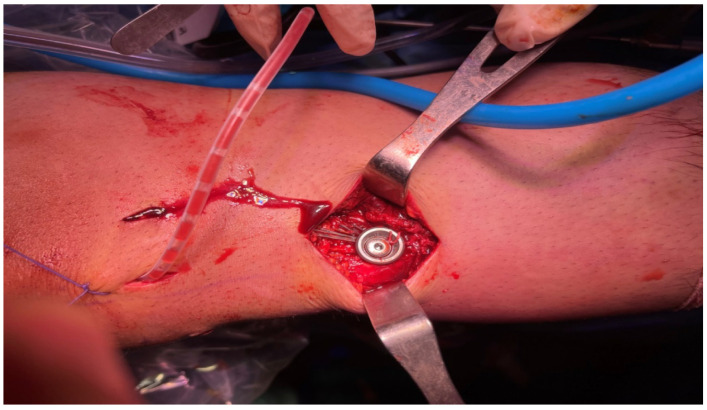
Tibial fixation of the tension sutures with a washer screw during anterior cruciate ligament reconstruction.

**Figure 8:**
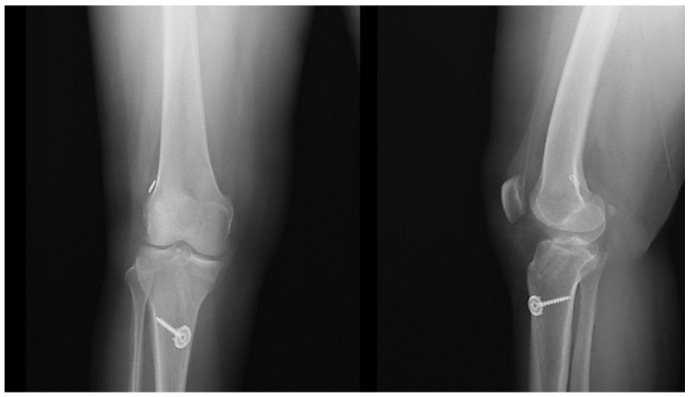
Postoperative anteroposterior and lateral radiographs demonstrating tibial fixation after anterior cruciate ligament reconstruction.

The tendons were cleansed in both groups, and their lengths were measured from end to end (Figure 4). Based on tendon length, the tendons were fixed to the EndoButton implant in 4-, 5-, or 6-fold configurations, and hamstring autografts were created (Figure 5). The tunnels in the femur and tibia were then opened. The graft was placed in the femoral and tibial tunnels. The EndoButton implant was used for femur fixation. The knee was stretched with the help of reinforced sutures at 20 degrees of flexion and fixed with a bioabsorbable screw 1 cm thicker than the tibial tendon. Tension threads consisting of reinforced sutures according to the length of the graft were fixed to the screw with scales, or, if the graft was long, it was fixed to the bone with staples (Figure 6 and Figure 7). Knee stability was examined, and a Hemovac drain was inserted. After the wounds were stitched up, the procedure was finished. A locking knee brace with an adjustable angle was put on each patient. All patients had their postoperative roentgenograms taken (Figure 8).

The IBM Statistical Package for the Social Sciences (SPSS), version 25.0 (IBM Corp., Armonk, NY, USA), was used to analyze the data. To evaluate whether the data fit a normal distribution, the Kolmogorov–Smirnov test was used. For comparison tests, the significance threshold (*p*) was set at 0.05. Because the data had a normal distribution, the significance of the difference between the two means was assessed using the significance test (*t*-test). We generated cross-tabulations and conducted a chi-square (χ^2^) analysis to analyze the categorical data. Multivariate logistic regression analysis was not performed in this study because the primary outcome variable (tendon length) was continuous.

## 3. Results

The demographic information, including age and BMI, for each patient participating in the study is included in Table 1 and Table 2. No statistically significant difference was found between Groups 1 and 2 in terms of gender, side, age, thigh circumference, BMI, or leg length (*p* > 0.05, Table 1 and Table 2).

The Cohen’s d values confirm this because they all fall into the “small” or “negligible” categories (typically d < 0.2).

**Figure 9:**
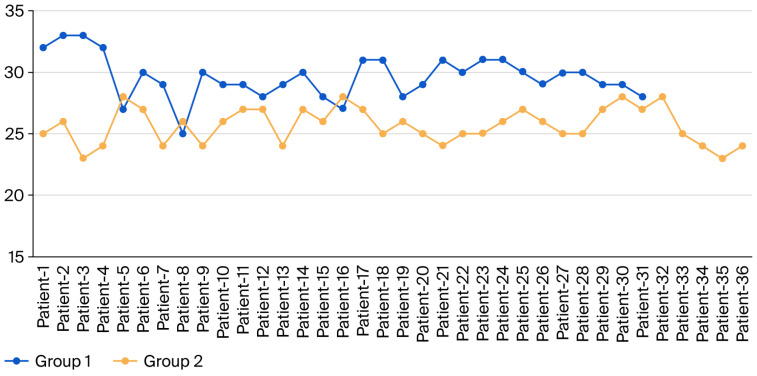
Semitendinosus tendon length distribution of the groups. The blue line indicates Group 1, and the red line indicates Group 2.

**Figure 10:**
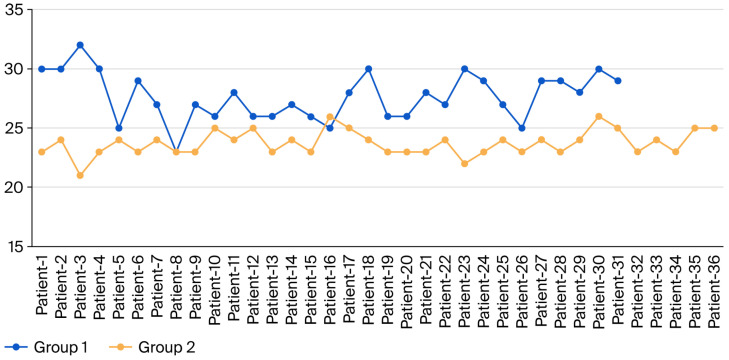
Gracilis tendon length distribution of the groups. The blue line indicates Group 1, and the red line indicates Group 2.

The semitendinosus tendon length (STL) and gracilis tendon length (GTL) of the study participants were compared to determine whether there was a difference between Groups 1 and 2. The results are presented in Table 3. The semitendinosus and gracilis tendon lengths in Group 1 were 29.61 ± 1.76 cm and 27.68 ± 2.02 cm, respectively. In Group 2, the semitendinosus and gracilis tendon lengths were 25.67 ± 1.45 cm and 23.72 ± 1.06 cm, respectively. A statistically significant difference in STL and GTL was observed between Groups 1 and 2 (*p* < 0.05, Table 3). Patients in Group 1 had longer STL and GTL than those who underwent tendon harvest with tourniquet use (Group 2) (Figure 9 and Figure 10).

The elevated Cohen’s d values demonstrate that the magnitude of the observed difference between the groups is highly significant from a practical perspective.

## 4. Discussion

The primary goal of anterior cruciate ligament reconstruction is to restore knee stability, reduce the risk of secondary meniscal tears and cartilage damage, and enable a return to pre-injury knee function. Multiple factors contribute to achieving this goal, among them graft characteristics such as quality, diameter, and configuration. Longer harvested tendons may allow greater flexibility in graft folding configurations, potentially influencing graft diameter. Adams and Magnussen reported higher revision rates with hamstring grafts < 8 mm in diameter [4,5]. In the study by Heijboer et al., the hamstring autograft diameter was shown to be influenced by the tendon morphology and graft preparation technique, with tendon length potentially playing a role in determining the achievable graft folding configuration [6]. In this context, the present study demonstrated that harvesting hamstring tendons without tourniquet use was associated with significantly longer tendon lengths, suggesting a potential technical factor that may influence graft preparation. Hamstring tendons are among the most commonly used autograft options for anterior cruciate ligament reconstruction due to advantages such as ease of access and harvest, the creation of a soft tissue tunnel, and the ability to achieve patient-specific graft length and diameter, compared with bone–patellar, tendon–bone, and quadriceps tendon grafts. Therefore, the use of hamstring tendons in all patients in the present study and the evaluation of surgical factors that may influence harvested tendon length can be considered clinically relevant.

A tourniquet is routinely applied to the proximal thigh during anterior cruciate ligament reconstruction to obtain a bloodless surgical field and improve intraoperative visualization [7,8]. However, arthroscopic ACL reconstruction is a relatively lengthy procedure, and tourniquet use has been associated with adverse effects, including muscle atrophy, metabolic changes, and postoperative discomfort [9,10]. Numerous studies have investigated the effects of tourniquet use during ACL reconstruction [11,12,13,14]. Yaghmour et al. evaluated 26 ACL reconstructions performed without tourniquet use and reported that tourniquet-less reconstruction was a feasible and effective alternative, with favorable outcomes for pain, knee swelling, range of motion, and bleeding [14]. Additional reported advantages of avoiding tourniquet use include earlier recovery of quadriceps muscle strength, reduced thigh atrophy, decreased postoperative pain, and fewer electromyographic changes, while tourniquet use has also been associated with tibial, femoral, and saphenous nerve palsy [11,12,13]. Yang et al. demonstrated that tourniquet use during ACL reconstruction increased postoperative pain and hemarthrosis and had a temporary negative effect on muscle strength [15]. Previous systematic reviews have evaluated the clinical effects of tourniquet use during anterior cruciate ligament reconstruction and reported that tourniquet application does not adversely affect long-term functional outcomes, although it may influence early postoperative pain and muscle function [16]. Despite extensive evaluation of multiple outcomes related to tourniquet use, data specifically addressing its effect on harvested hamstring tendon length remain limited. In the present study, hamstring tendons harvested without tourniquet use were significantly longer than those obtained with tourniquet application. Therefore, avoiding tourniquet use during hamstring tendon harvest may represent a technical advantage by increasing harvested tendon length during ACL reconstruction (Table 3).

Numerous studies have evaluated various parameters related to hamstring tendon grafts in anterior cruciate ligament reconstruction. Gupta et al. investigated four-strand hamstring tendon grafts and reported that grafts with a diameter smaller than 8 mm were associated with an increased risk of ACL graft rupture, whereas grafts with a diameter greater than 8 mm were associated with improved knee stability and higher rates of return to sport [17]. Krishna et al. demonstrated that a five-strand graft configuration was associated with better clinical outcomes, whereas a four-strand configuration was frequently associated with a graft diameter < 8 mm [18]. Francisco et al. emphasized that hamstring autograft size is a clinically relevant factor in ACL reconstruction outcomes. Although the optimal graft diameter for preventing failure has not been definitively established, recent evidence suggests that incremental increases of approximately 0.5 mm up to 10 mm may be beneficial. However, there is currently no evidence supporting the routine use of grafts larger than 10 mm, indicating that graft size alone should not be the sole objective of ACL reconstruction [19]. In this context, the findings of the present study suggest that harvested tendon length may be a clinically relevant factor, providing greater flexibility in graft folding configurations during ACL reconstruction. Recent literature has increasingly focused on graft preparation techniques and their biomechanical implications in anterior cruciate ligament reconstruction. Various hamstring graft configurations have been described, including four-strand, five-strand, tripled semitendinosus, and quadrupled semitendinosus constructs, which are used to optimize graft diameter and biomechanical strength. Rovere et al. emphasized that graft preparation strategies often depend on the available tendon length and surgeon preference, highlighting that adequate tendon length is a critical prerequisite for more complex graft constructs. In this context, longer harvested tendons may provide greater flexibility during graft preparation, allowing surgeons to choose alternative graft configurations that may improve graft diameter and biomechanical stability. Therefore, the increased tendon length observed in the present study when a tourniquet was not used may have practical relevance beyond a purely intraoperative observation because it may facilitate the creation of different graft constructs and potentially contribute to improved graft characteristics in ACL reconstruction [20]. A clinically important aspect of tendon length during ACL reconstruction is its influence on graft preparation strategies. When the harvested tendon is relatively short, surgeons may have a limited number of strands to use for graft preparation, which may restrict the ability to achieve an optimal graft diameter. In contrast, longer harvested tendons provide greater flexibility in graft configuration, allowing the use of alternative constructs such as five- or six-strand hamstring grafts when necessary. Although graft diameter and biomechanical properties were not directly evaluated in the present study, the observed difference in tendon length may still have practical implications for intraoperative decision-making, particularly in patients with relatively small hamstring tendons. Therefore, increased tendon length may represent a technical advantage that facilitates graft preparation rather than a direct determinant of clinical outcomes.

The length and thickness of hamstring tendons have been investigated in several preoperative studies. Hollnagel et al. reported that magnetic resonance imaging (MRI) can be used to preoperatively estimate hamstring autograft size for anterior cruciate ligament reconstruction and may assist orthopedic surgeons in graft selection and surgical planning in clinical practice [21]. Purohit et al. found that the preoperative prediction of graft parameters was clinically meaningful, reporting that patient weight and thigh length contributed to the estimation of graft diameter and length [22]. Furthermore, male patients with a height < 155 cm, body weight < 58 kg, and thigh length < 40 cm were found to be at higher risk of receiving thinner grafts and shorter tendon lengths [23]. Although accurate prediction remains challenging, hamstring autograft diameter has been reported to be associated with patient sex, height, body mass index, and thigh circumference [22,24]. Knowledge of factors such as height, age, and body weight may therefore aid in the preoperative estimation of graft diameter and appropriate graft selection [24]. Mishra et al. reported that patient height was associated with semitendinosus graft length and diameter, independent of lower extremity activity level [25].

In addition, several surgical techniques have been described to obtain longer hamstring tendons. Frank et al. emphasized the meticulous dissection of accessory structures and fascial bands before using tendon strippers to avoid premature tendon transection and facilitate the harvest of longer hamstring grafts [26]. The authors noted that early transection was unlikely if a tendon extension of 10–12 cm was preserved for each tendon [26]. Candal-Couto and Deehan conducted an anatomical study on ten cadaveric specimens to characterize the fascial attachments of the gracilis and semitendinosus tendons, reporting considerable variability in accessory bands, including intertendinous connections and attachments to the popliteal fascia, sartorius, gastrocnemius, pretibial fascia, and superficial fascia. The release of these accessory bands was considered critical during tendon harvest [27]. Joshi et al. reported that the presence of accessory structures or bands around the knee may complicate routine harvesting of the gracilis and semitendinosus tendons [28]. Tuncay et al. identified differences in the size and location of fascial bands between the semitendinosus and gracilis tendons as a primary cause of premature tendon transection [29]. Yiannakopoulos et al. stated that the success of anterior cruciate ligament reconstruction largely depends on preservation of the length and integrity of the hamstring tendons and therefore developed an endoscopic-assisted tendon harvesting technique to achieve this goal [30]. Çatma et al. investigated the effect of a wide-contour pneumatic tourniquet (PT) and an ultra-narrow sterile elastic tourniquet (SET) on the length of hamstring autografts used for anterior cruciate ligament reconstruction in patients with tubular and conical femoral morphologies. They reported that using SET, which occupies less space on the thigh, resulted in significantly longer hamstring autografts than PT and that femoral graft length became largely independent of the conicity index when SET was used [31]. Collectively, these findings indicate that numerous approaches have been explored to assess and optimize hamstring tendon size and length. In this context, the present study demonstrated that avoiding tourniquet use during the intraoperative period was associated with significantly longer hamstring tendon harvests (Table 3). The findings of the present study may encourage further biomechanical and mechanistic investigations to better understand the factors influencing harvested hamstring tendon length during ACL reconstruction. Although the present study does not definitively explain the underlying mechanism, it highlights a potentially relevant surgical factor and may open new avenues for future research in this area. A possible explanation for the observed difference in tendon length may relate to the mechanical effects of tourniquet compression on the surrounding soft tissues. Inflation of a proximal thigh tourniquet may increase local soft tissue pressure and alter the tension of the pes anserinus region, potentially limiting tendon excursion during harvesting. In addition, compression of the soft tissues around the medial knee may influence the identification and release of accessory fascial bands that are known to affect successful hamstring tendon harvesting. It should also be acknowledged that the observed difference in tendon length may not be attributable solely to tourniquet inflation. Surgical handling, tissue tension, limb positioning, and other subtle technical factors during tendon harvesting may also influence the effective length of the harvested tendon.

One of the limitations of this study is the relatively small sample size. Another limitation of the present study is that only tendon length was evaluated as the primary outcome. Parameters such as final graft diameter, graft configuration, operative time, blood loss, postoperative pain, and early functional outcomes were not assessed. Future studies evaluating these parameters may help clarify the clinical implications of increased hamstring tendon length during ACL reconstruction.

## 5. Conclusions

These findings suggest that avoiding tourniquet use during hamstring tendon harvest may represent a simple technical modification that can influence harvested tendon length during anterior cruciate ligament reconstruction.

## Figures and Tables

**Table 1:** Distribution of patients according to sex and surgical side.

Variable		Group 1 (*n* = 31)	Group 2 (*n* = 36)	Total (*n* = 67)	χ^2^	*p* Value
Sex	Male	25 (80.65%)	27 (75.00%)	52 (77.61%)	0.305	0.580
	Female	6 (19.35%)	9 (25.00%)	15 (22.39%)		
Side	Right	20 (64.52%)	26 (72.22%)	46 (68.66%)	0.460	0.498
	Left	11 (35.48%)	10 (27.78%)	21 (31.34%)		

*n*: number of samples; %: percent; χ^2^: Chi-square Test value.

**Table 2:** Demographic and anthropometric characteristics of the patients.

Variable	Group 1 (Mean ± SD)	Group 2 (Mean ± SD)	*t*	*p* Value	%95 CI		Cohen’s d
					Lower	Upper	
Age (years)	31.35 ± 9.07	33.42 ± 8.88	−0.938	0.352	−6.450	2.327	−0.231
BMI (kg/m^2^)	24.52 ± 1.79	24.31 ± 1.60	0.509	0.613	−0.616	1.037	0.124
Leg length (cm)	84.84 ± 3.87	84.69 ± 3.79	0.154	0.878	−1.730	2.019	0.039
Thigh circumference (cm)	47.39 ± 1.61	47.31 ± 1.70	0.201	0.842	−0.730	0.894	0.048

BMI: Body mass index; SD: standard deviation; *t*: independent-samples *t* test; *p* value: statistical significance; CI: confidence interval; *p* < 0.05.

**Table 3:** Comparison of hamstring tendon lengths between groups.

Variable	Group	Mean ± SD	Min–Max	*t*	*p* Value	%95 CI		Cohen’s d
						Lower	Upper	
Semitendinosus tendon length (cm)	Group 1	29.61 ± 1.76	25–33	10.037	<0.001 *	3.161	4.731	2.44
	Group 2	25.67 ± 1.45	23–28					
Gracilis tendon length (cm)	Group 1	27.68 ± 2.02	23–30	10.225	<0.001 *	3.183	4.728	2.46
	Group 2	23.72 ± 1.06	21–26					

Min–Max: Minimum–maximum; cm: centimeters; SD: standard deviation; *t*: independent-samples *t* test; *p* value: statistical significance; CI: confidence interval; * *p* < 0.05.

## Data Availability

The datasets used and/or analyzed during the current study are available from the corresponding author on reasonable request.
